# A Neural Network Model for *K*(*λ*) Retrieval and Application to Global *K*_par_ Monitoring

**DOI:** 10.1371/journal.pone.0127514

**Published:** 2015-06-17

**Authors:** Jun Chen, Yuanli Zhu, Yongsheng Wu, Tingwei Cui, Joji Ishizaka, Yongtao Ju

**Affiliations:** 1 Key Laboratory of Coastal Wetland Biogeosciences of China Geological Survey, Qingdao Institute of Marine Geology, Qingdao, 266071, China; 2 Hydrospheric Atmospheric Research Center, Nagoya University, Nagoya, 4648601, Japan; 3 Graduate School of Environmental Studies, Nagoya University, Nagoya 4648601, Japan; 4 Bedford Institute of Oceanography, Fisheries and Oceans Canada, Dartmouth, NS, B2Y4A2, Canada; 5 First Institute of Oceanography, State Oceanic Administration, Qingdao, 266071, China; 6 College of Mining Engineering, Hebei United University, Tangshan 063009, China

## Abstract

Accurate estimation of diffuse attenuation coefficients in the visible wavelengths *K*_d_(*λ*) from remotely sensed data is particularly challenging in global oceanic and coastal waters. The objectives of the present study are to evaluate the applicability of a semi-analytical *K*_d_(*λ*) retrieval model (SAKM) and Jamet’s neural network model (JNNM), and then develop a new neural network *K*_d_(*λ*) retrieval model (NNKM). Based on the comparison of *K*_d_(*λ*) predicted by these models with in situ measurements taken from the global oceanic and coastal waters, all of the NNKM, SAKM, and JNNM models work well in *K*_d_(*λ*) retrievals, but the NNKM model works more stable and accurate than both SAKM and JNNM models. The near-infrared band-based and shortwave infrared band-based combined model is used to remove the atmospheric effects on MODIS data. The *K*_d_(*λ*) data was determined from the atmospheric corrected MODIS data using the NNKM, JNNM, and SAKM models. The results show that the NNKM model produces <30% uncertainty in deriving *K*_d_(*λ*) from global oceanic and coastal waters, which is 4.88-17.18% more accurate than SAKM and JNNM models. Furthermore, we employ an empirical approach to calculate *K*_par_ from the NNKM model-derived diffuse attenuation coefficient at visible bands (443, 488, 555, and 667 nm). The results show that our model presents a satisfactory performance in deriving *K*_par_ from the global oceanic and coastal waters with 20.2% uncertainty. The *K*_par_ are quantified from MODIS data atmospheric correction using our model. Comparing with field measurements, our model produces ~31.0% uncertainty in deriving *K*_par_ from Bohai Sea. Finally, the applicability of our model for general oceanographic studies is briefly illuminated by applying it to climatological monthly mean remote sensing reflectance for time ranging from July, 2002- July 2014 at the global scale. The results indicate that the high *K*_d_(*λ*) and *K*_par_ values are usually found around the coastal zones in the high latitude regions, while low *K*_d_(*λ*) and *K*_par_ values are usually found in the open oceans around the low-latitude regions. These results could improve our knowledge about the light field under waters at either the global or basin scales, and be potentially used into general circulation models to estimate the heat flux between atmosphere and ocean.

## Introduction

Sunlight provides the major energy source fueling for marine ecology in our blue planet. Due to the fact that all benthic substrates should receive enough light to sustain photosynthesis for primary production (conspicuous as sea grasses [1], algae and corals, and less conspicuous such as the microflora thriving in sandy and muddy bottoms [2]), the light in the shallow ocean should receive much more attenuation than it presently doses. Traditionally, the light available in the water column in the visible parts of the spectrum (400–700 nm) is usually defined as photosynthetically active radiation (PAR) [3, 4]. As a natural component of irradiance arriving at the Earth, the PAR is an important factor that could influence the ecological processes, heat budgets, and biogeochemical cycles in the upper layer of oceans [5, 6]. Generally, the PAR attenuation is quantified as the diffuse attenuation coefficient of the downwelling spectral irradiance (*K*_d_(*λ*), where *λ* refers to wavelength) at wavelength 490 nm [3, 7]. However, due to the fact that longer wavelengths are disproportionately absorbed in near-surface waters [8], using *K*_d_(*λ*) at a single band yields a poor approximation of PAR in the upper layers of oceans [9]. To account for this, the spectral *K*_d_(*λ*) should be known in the future.

At present there is a compelling need for a spectral *K*_d_(*λ*) model for turbid waters, as the existing models are essentially applicable only for *K*_d_(488) [10–12]. Recently, based on the radiative transfer equation (RTE), Lee et al. [8] developed a semi-analytical *K*_d_(*λ*) retrieval model (SAKM) that can retrieve *K*_d_(*λ*) from the known spectral absorption (*a*(*λ*)) and backscattering (*b*_*b*_(*λ*)) coefficients. The model was further evaluated by dataset simulated using the widely accepted numerical code with input bio-optical conditions generated based on extensive measurements made in the field [13], indicating that the model-derived *K*_d_(*λ*) matched with the simulation results very accurately. However, the retrieval accuracy of SAKM model strongly depends on the estimation accuracy of quasi-analytical model (QAA)-derived *a*(*λ*) and *b*_*b*_(*λ*) [8]. Actually, the QAA model is able to suppress the effect of total backscattering coefficient, *b*_b_(*λ*), instead of eradicating it completely [14], which in turn lead to the violation of QAA model in turbid coastal waters. Moreover, Wang et al. [10] and Chen et al. [11] also suggested that some problems may be encountered, when the SAKM model is used to derive *K*_d_(*λ*) from turbid coastal waters. Therefore, a model which is capable of providing a higher level of accuracy for *K*_d_(*λ*) estimation remains desired.

As the purely radiative transfer approach is hindered by the rigorous model’s inputs such as the profile information of the inherent optical properties of the water and atmosphere, the empirical methods are still a good choice if the optical behaviors of optically activity constituents could be assimilated into the models [15]. One possible method in this direction is the application of a neural network *K*_d_(*λ*) retrieval model (NNKM). For example, Jamet et al. [16] reported that the *K*_d_(*λ*) could be retrieved from the remote sensing reflectance at MODIS bands ranging from 412 to 667 nm using neural network model (JNNM). Unfortunately, MODIS sensor has calibration and atmospheric correction problems at 412 nm [17, 18], which in turn decreases the accuracy and stability of JNNM model. Therefore, to improve the performance of neural network models, it is necessary to optimize the model’s inputs.

The distribution of the PAR under sea-surface is mainly controlled by the waters’ optical behaviors expressed through the diffuse attenuation coefficient (*K*_par_), so the accurate estimation of *K*_par_ in the water column is critical for understanding the linkage between the physical processes and biological processes in the euphotic zone [3, 19]. From an optical perspective, in addition to water molecules, light attenuation is mainly constrained by the concentration of three independent optically activity constituents [20]: pigment, dissolved organic matter (CDOM) and suspended particulate matters (SPM). Traditionally, the *K*_par_ was measured by the ocean color scientific community at 490 nm (*K*_490_) and the following primary study was investigated in 1970s [21]. Due to the wavelength selected absorbing characteristics of the optically activity constituents [8, 22], once PAR penetrates into the sea, the spectral shape changes with the increase of depth. For example, the PAR is easy to penetrate below the surface, but undergoes a shift from the blue-green spectrum (400–500 nm) in open ocean waters to the green spectrum (500–550 nm) in coastal waters, according to increasing water column turbidity [9, 23]. Ecologically speaking, the maximum optical depth at which phytoplankton can photosynthesize is defined conventionally as 1–0.1% of the surface light [24], regardless of the longer wavelength, red in this example, is rapidly attenuated in the water column relatively to the short wavelength blue even for well-mixed waters [19], which in turn lead to the underestimation of PAR under ocean-surface using the traditional *K*_490_-*K*_par_ retrieval model.

For the estimation of *K*_d_(*λ*) and *K*_par_, a model which is capable of providing a higher level of accuracy remains desired. The specific goals of the study are as follows: (1) to assess the accuracy of SAKM and JNNM models in retrieving *K*_d_(*λ*) from the global oceanic and coastal waters; (2) to improve the performance of SAKM and JNNM models by proposing the NNKM model; (3) to develop an innovative NNKM-extended *K*_par_ retrieval model (NKKM); and (4) to depict the spatial and temporal variation of *K*_d_(*λ*) and *K*_par_ in global oceanic and coastal waters. The major difference between this study and previous reports lies in that the spectral *K*_d_(*λ*) in the visible regions is developed and applied for retrieving *K*_par_ from the global oceanic and coastal waters.

## Data and Methods

### Datasets used

A dataset consisting of 1873 paired (Tables 1 and 2), in situ measurements of multispectral *R*_rs_(*λ*) and *K*_d_(*λ*) from diverse water types was used to train and test the accuracy and stability of SAKM, JNNM, and NNKM models in deriving *K*_d_(*λ*) from the global oceanic and coastal waters. These data came from various researchers around the United States and Europe and contain mostly subsurface values of *R*_rs_(*λ*) and *K*_d_(*λ*). The training dataset was measured from two measurement subsets collected by two independent research teams. The first subset (*n* = 1837) was achieved by the NASA SeaWIFS Project as the NOMAD dataset [25], while the second subset (*n* = 125) was collected from Bohai Sea during 2005 [11]. The testing dataset was also measured from two measurement subsets provided by another two independent research teams.

**Table 1:** Descriptive statistics of the field-measured *K*_d_(*λ*) in training dataset, 1962 samples.

a. Calibration dataset taken from global oceanic and coastal waters, 1962 samples					
	Min	Max	Median	Average	SD
*K*_d_(443), m^-1^	0.009	4.114	0.072	0.201	0.417
*K*_d_(488), m^-1^	0.009	3.659	0.058	0.152	0.326
*K*_d_(555), m^-1^	0.025	3.311	0.009	0.152	0.240
*K*_d_(667), m^-1^	0.016	3.941	0.560	0.658	0.351

**Table 2:** Descriptive statistics of the field-measured *K*_d_(*λ*) in testing dataset, 174 samples.

	Min	Max	Median	Average	SD
*K*_d_(443), m^-1^	0.035	2.338	0.229	0.350	0.442
*K*_d_(488), m^-1^	0.023	1.948	0.152	0.257	0.353
*K*_d_(555), m^-1^	0.064	1.426	0.138	0.227	0.260
*K*_d_(667), m^-1^	0.299	1.798	0.507	0.609	0.271

To evaluate the accuracy of NKKM model in quantifying *K*_par_, 664 samples consisting of remote sensing reflectance (*R*_rs_(*λ*)) and *K*_par_ was obtained from NOMAD dataset. In addition to these data, we collected 105 data points from China East Sea, China, 17 data points from Chesapeake Bay, USA, 21 data points from Ariake Bay, Japan, and 26 samples collected from Bohai Sea, China. All of these datasets were consisting of synchronous *R*_rs_(*λ*) and *K*_par_ measurements from above-surface and/or subsurface. These data were measured by various researchers around United States, Europe, and Asia using various instrumentations, with all measurements closely following rigorous and community-defined deployment and data processing protocols. To evaluate the accuracy of satellite-*K*_par_, three MODIS imageries scanned over Bohai Sea on September 1, 14, and 22 were collected in this study. These MODIS data were synchronized with the Bohai Sea dataset. Finally, the five independent datasets (Table 3) were divided into three groups: (1) the calibration dataset containing 664 samples, taken from NOMAD; (2) the validation dataset consisting 143 samples, taken from the China East Sea, Chesapeake Bay, and Ariake Bay; and (3) the match-up analysis dataset, consisting 26 samples taken from the Bohai Sea.

**Table 3:** Descriptive statistics of the measured *K*_par_ (m^-1^) taken in NOMAD, China East Sea, Chesapeake Bay, and Ariake Bay, where Std refers to the standard deviation.

Dataset	Min	Max	Median	Mean	Std
NOMAD	0.005	0.825	0.077	0.127	0.125
China East Sea	0.058	0.342	0.167	0.171	0.058
Chesapeake Bay	0.403	0.792	0.670	0.611	0.142
Ariake Bay	0.284	2.176	0.427	0.546	0.402
Bohai	0.238	2.175	0.582	0.924	0.653

### Construction of NNKM model

If properly initialized, the neural network model was able to yield accurate retrieval of *K*_d_(*λ*) in the turbid coastal waters [16, 26, 27]. A typical structure of neural network model includes an input layer, one or more hidden layers and an output layer. The input layer only distributes the input signals into the network, without processing them, but the nodes in the hidden layers and in the output layer transform their input signal by an activation function. Therefore, the neural network with no hidden layers could only be used to simulate the linear relationships, while a single hidden layer with adequate nodes allowed the approximation of any function that contained a continuous mapping from one finite space to another. Only for some particularly complicated cases, two hidden layers were used, but there was absolutely no theoretical reason to use more than two hidden layers, because more hidden layers would make the neural networks be more prone to over fitting the data. Once the architecture of neural network model is designed, the relationship between the inputs and outputs ultimately depends on the weight values of the nodes. Generally, the weight values are determined by the supervised learning technique.

**Fig 1:**
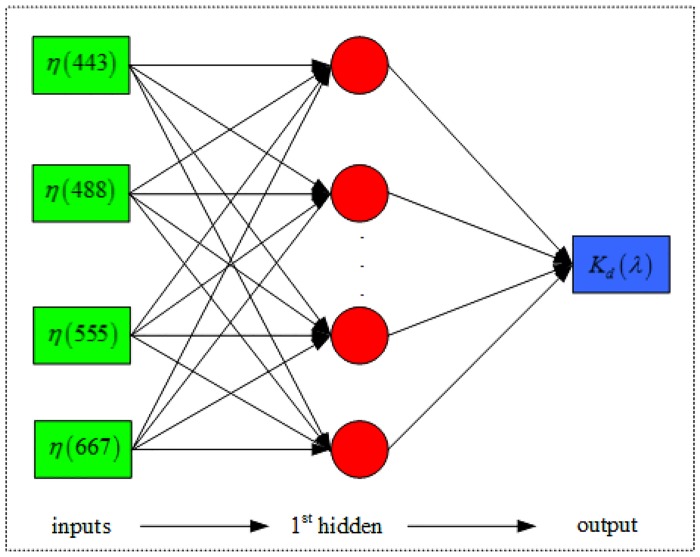
Basic architecture of the NNKM model used in this work.

It was well known that the increasing number of inputting parameters would result in an increase of the “degree-freedom” of the model, which in turn may decrease the stability and accuracy of the neural network model [28]. Therefore, it was necessary to optimize the inputs of neural network models. As [29] and Lee et al. [8] indicated that *K*_d_(*λ*) could be denoted as a function of *a*(*λ*) and *b*_b_(*λ*). Recently, Chen [14] suggested that *a*(*λ*) and *b*_b_(*λ*) could be retrieved from logarithmic values of remote sensing reflectance (*η*(*λ*)) at 443, 488, 555, and 667 nm after removing the Raman scattering effects. Moreover, many literatures [10–12, 30–32] reported that *R*_rs_(667)/*R*_rs_(488) works better in turbid coastal waters, while *R*_rs_(555)/*R*_rs_(488) is more effectively in opening oceanic waters for *K*_d_(490) estimations. Binding et al. [33] and D’Sa et al. [34] proposed that red/green ratios work well in accounting for variations of suspended particulate matters in oceanic waters. These findings implied that the *η*(*λ*) at 443, 488, 555, and 667 nm could be used to construct the NNKM model for *K*_d_(*λ*) retrieval. Therefore, the basic architecture as shown in Fig 1 is used to establish the NNKM model for estimations of *K*_d_(*λ*).

The preparation of model inputs and determination of model architecture and parameters are two important steps to model and simulate the *K*_d_(*λ*) concentration in the global oceanic and coastal waters using the NNKM model. The network was initialized using a training dataset (Table 1). The weight values of NNKM model were calculated by a supervised learning approach, using a priori information about the actual output which corresponds to a set of inputs. A back propagation learning procedure was used to iteratively compute the optimal weights for neural network. The conjugate-gradient technique, an iterative optimization method adapted to multi-layer perceptron, was used as the back-propagation gradient. The activation function for hidden layers was a nonlinear hyperbolic tangent function, while the output node was only applied with a bias transfer function. The architecture contained one hidden layer with number of nodes varying from 1 to 50 was tested to find out the optimal architecture for NNKM model. Overall considering the MRE value and number of neurons, the optimal architecture was therefore composed of one hidden layer with 9 neurons.

### Establishment of NKKM model

The *K*_d_(*λ*) is the coefficient of the exponential attenuation of the spectral downward irradiance [20]:

$$E_{d}\left(\lambda ,z\right)=E_{d}\left(\lambda ,z=0\right)Exp\left[-K_{d}\left(\lambda \right)z\right]$$ (1)

Where, *E*_*d*_(*λ*,*z*) is the downward spectral solar irradiance at wavelength *λ* and depth *z*. If the visible spectral domain is considered, the PAR at depth *z* is traditionally defined as [35]:

$$PAR\left(z\right)=\int_{400}^{700}E\left(\lambda ,z=0\right)Exp\left[-K_{d}\left(\lambda \right)z\right]d\lambda$$ (2)

Following the scheme of Eq (1), the vertical propagation of PAR is also commonly defined as:

$$K_{par}=-\frac{\text{ln}\left[PAR\left(z\right)\right]-\text{ln}\left[PAR\left(0\right)\right]}{z}$$ (3)

Substitute Eq (2) into Eq (3), yields:

$$K_{par}=-\frac{\text{ln}\left\{\int_{400}^{700}w_{E}Exp\left[-K_{d}\left(\lambda \right)z\right]d\lambda \right\}}{z}$$ (4)

Where, *w*_E_ refers to the ratio of solar irradiance at wavelength *λ* to the PAR just below the sea-surface, whose value can be calculated using *E*(*λ*)/*PAR*(0). It is very difficult to find out a general solution for *K*_par_ in Eq (4), because this general solution not only relates to the variations of PAR at the top-of-atmosphere [35], but also associates with atmospheric optical behaviors such as light absorption and scattering [36]. However, Following Eq (4), we could know that the *K*_par_ is a function of *K*_*λ*_. For simplicity, we assume that the *K*_par_ may be approximately expressed as:

$$K_{par}=\sum_{i=1}^{n}w_{i}K_{\lambda_{i}}^{v_{i}}$$ (5)

Where, *w*_i_ and *v*_i_ are the empirical coefficients at the *i* ^th^ wavelength. General speaking, no simplified assumption can be made that is valid for all special cases existing in the natural world, so no universal model can be used to describe the relationship between remote sensing reflectance and *K*_*λ*_ with no uncertainty. These uncertainties will be inevitable to transfer from the calculated *K*_d_(*λ*) to *K*_par_, which in turn lead to the model-derived *K*_par_ containing some systematic and/or random noises due to the inaccuracy *K*_d_(*λ*) retrieval results. Fortunately, it is expected that the accuracy of *K*_par_ retrieval model may be improved if the *K*_d_(*λ*) and *K*_par_ estimations were combined together, because some noise generated in the *K*_d_(*λ*) retrieval step could be minimized during the procedures of the *K*_par_ model initialization. Therefore, in this study, we used the neural network model-derived *K*_d_(*λ*) synchronizing with field-measured *K*_par_ data to initialize the NKKM model as shown in Eq (5).

### Accuracy assessment

In this study, the root-mean-square of the ratio of the modeled-to-measured values is used to assess the accuracy of the atmospheric correction. This statistic will be referred to as *MRE* and is described by the following equation [11, 37, 38]:

$$ARE_{i}=\left|\frac{x_{\text{mod},i}-x_{obs,i}}{x_{obs,i}}\right|\times 100\%$$ (6)

$$MRE=\sqrt{\frac{1}{n}\sum_{i=1}^{n}ARE_{i}^{2}}\times 100\%$$ (7)

Where, *ARE*_*i*_ is the relative uncertainty of the *i* ^th^ observation, *x*_mod,i_ is the modeled value of the *i* ^th^ element, *x*_obs,i_ is the observed value of the *i* ^th^ element, and *n* is the number of elements.

## Results

### Evaluation of SAKM and JNNM models

SAKM and JNNM models have been described in detail in various references [8, 16, 39]. SAKM model is a global algorithm that has been initialized using the global dataset [8, 40], so the coefficients of this model would not be adjusted according to the bio-optical dataset collected in this study here. It was noteworthy that the SAKM model-required solar zenith angle was computed for each station using information on time and location [41]. As Brewin et al. [41], Lee et al. [8], and Chen et al. [39] indicated that SAKM model produced reasonable performance against NOMAD data, only the weights of JNNM model were adjusted according to the training dataset as shown in Table 1. Since the MODIS sensor has calibration and atmospheric correction problems at 412 nm [17, 42, 43], the *K*_d_(412) retrieval accuracy would not be presented and discussed in this study.

**Fig 2:**
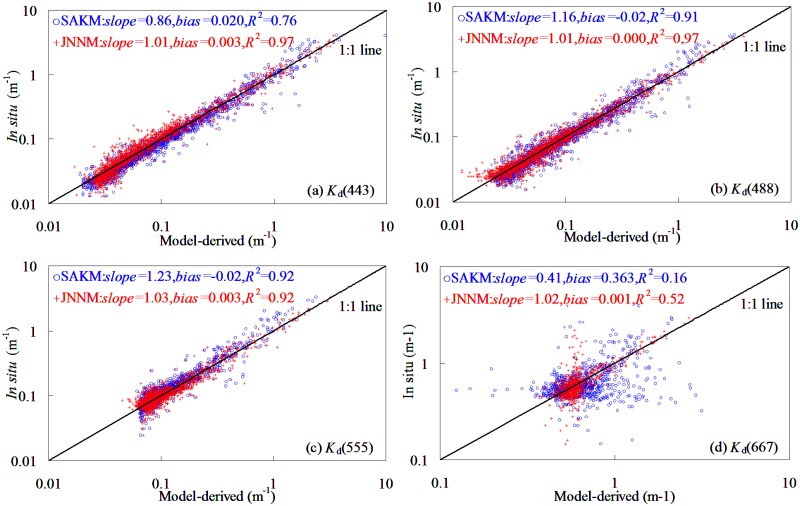
SAKM and JNNM models-derived plotting against field-measured *K*_d_(*λ*) in global oceanic and coastal waters,1962 samples (1837 points from NOMAD dataset and 125 points from Bohai Sea dataset).

The model’s evaluation was based on comparison of the *K*_d_(*λ*) predicted by SAKM and JNNM models with field-measured *K*_d_(*λ*), as shown in Fig 2. It was found that expect 667 nm band, both SAKM and JNNM models performed reasonably well in deriving *K*_d_(*λ*) from the global oceanic and coastal waters. For example (expect 667 nm band), For *K*_d_(*λ*) ranging from 0.009 to 4.114 m^-1^, the slopes of linear relationships between field-measured and model-predicted *K*_d_(*λ*) varied among 443–667 nm from 0.86 to 1.23, while the corresponding determination coefficients did not smaller than 0.52. The determination coefficients had a significant band changing pattern with values decreasing in the order of 555 nm>488 nm>443 nm>667 nm. Judging by determination coefficients, use of SAKM and JNNM models could account for 76–97% variations of *K*_d_(*λ*) in the global oceanic and coastal waters. These findings implied that both SAKM and JNNM models could be used to derive *K*_d_(*λ*) from global oceanic and coastal waters.

However, some limitations could also be encountered, when the SAKM and JNNM models were applied to retrieve *K*_d_(*λ*) from training dataset. For example, for very clearly waters and highly turbid waters, both SAKM and JNNM models became unstable and inaccurate. When using SAKM model, the *K*_d_(*λ*) was clearly overestimated in the low values (*K*_d_(*λ*)<0.4 m^-1^), while was significantly underestimated in the high values (*K*_d_(*λ*)>0.6 m^-1^). The determination coefficients of SAKM and JNNM models at 667 nm were very lower (<0.52), which may lead to the result that both models could not meet the requirements for *K*_d_(667) retrieval in the global oceanic and coastal waters.

### NNKM model training

**Fig 3:**
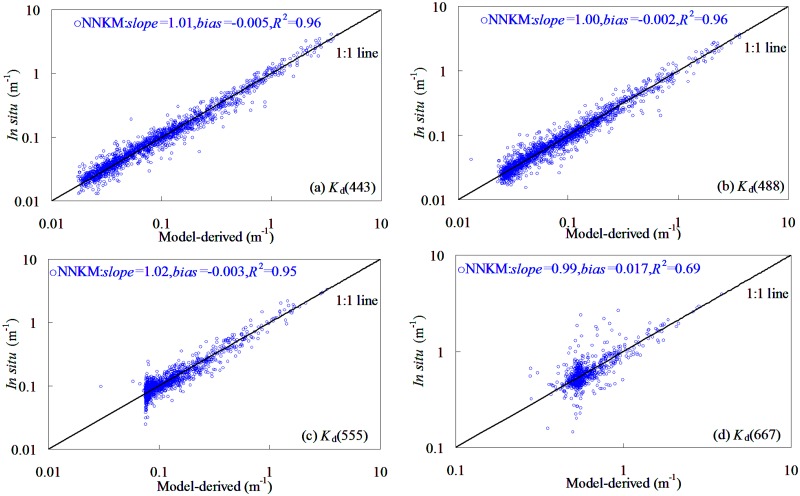
NNKM model-derived plotting against field-measured *K*_d_(*λ*) in global oceanic and coastal waters,1962 samples (1837 points from NOMAD dataset and 125 points from Bohai Sea dataset).

Based on 1962 field samples, the model shown in Fig 3 was proposed as the optimal NNKM model in quantifying *K*_d_(*λ*) from global oceanic and coastal waters. It was found that the new neural network model for retrieving *K*_d_(*λ*) performed reasonably well with the determination coefficients did not lower than 0.65. This was to say that use of NNKM model could account for >65% variations of *K*_d_(*λ*) in the training dataset. The determination coefficients had a significant band changing pattern with values decreasing in the order of 488 nm>443 nm>555 nm>667 nm. For training dataset, the slopes of linear relationships between field-measured and model-predicted *K*_d_(*λ*) varied among 443–667 nm from 1.0 to 1.17. Judging by determination coefficients, the NNKM model produced a superior performance to SAKM and JNNM models. However, Fig 3 also revealed that the scatter plots of field-measured versus NNKM model-derived *K*_d_(*λ*) at 667 nm was significantly dispersed from 1:1 line, even though the performance of SAKM and JNNM models in *K*_d_(667) estimations was pronounced improved by NNKM model. It seemed that the *K*_d_(*λ*) values at 667 nm were much more difficult to retrieve by SAKM, JNNM, and NNKM models than other wavelengths.

### NNKM model validation and comparison

**Fig 4:**
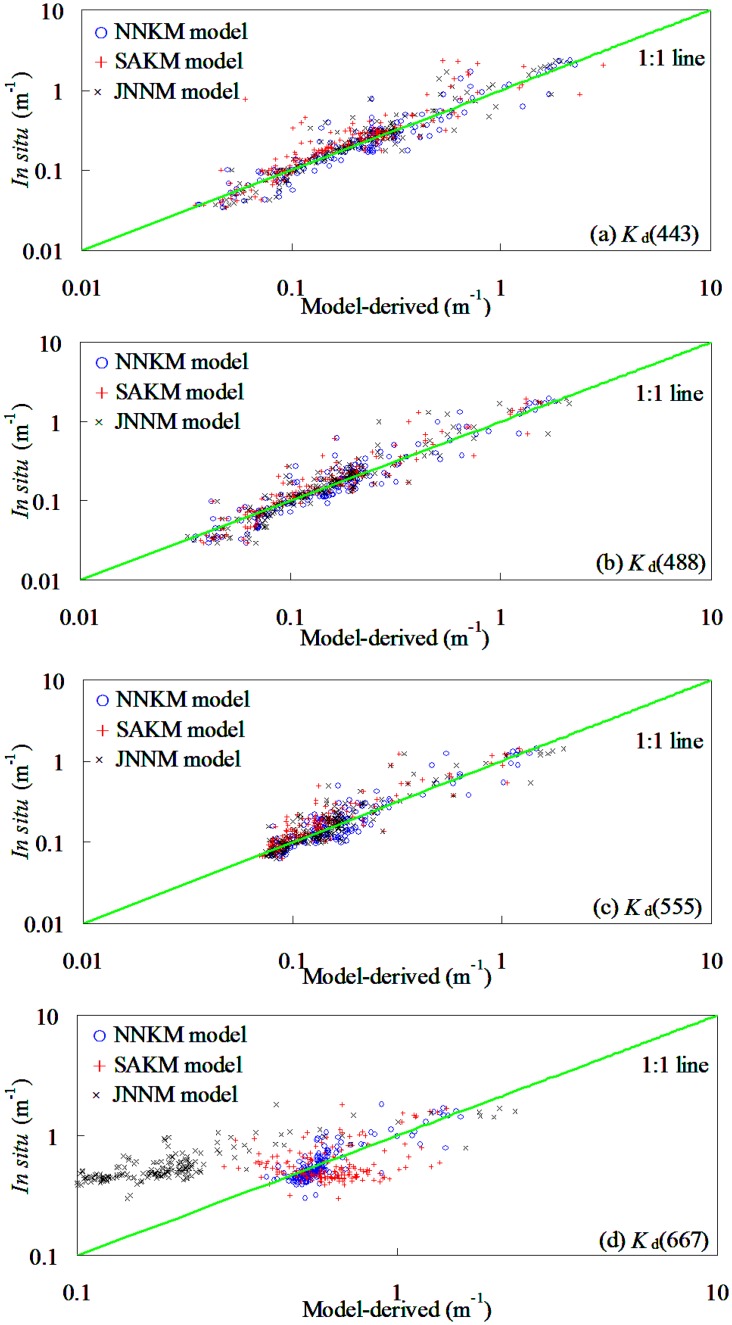
Comparison of the NNKM, SAKM, and JNNM models-derived *K*_d_(*λ*) with in situ measurements from Yellow Sea and China East Sea (174 samples).

To investigate whether or not the SAKM, JNNM, and NNKM models of *K*_d_(*λ*), developed for global oceanic and coastal waters, perform well in optical complicated shelf seas, the dataset taken from the Yellow Sea and East China Sea was used for model evaluations. Fig 4 and Table 4 showed the scatter plots of SAKM model-derived versus field-measured *K*_d_(*λ*) in testing dataset (Table 2). It was found that the NNKM model produced an acceptable accuracy (20.19%<*MRE*<29.17%) in deriving *K*_d_(*λ*) from the Yellow Sea and China East Sea, even though the field measurements in these shelf seas covered quite a wide variation of *K*_d_(*λ*) (e.g. 0.03 m^-1^<*K*_d_(490)<1.95 m^-1^). The slopes of linear relationship of field-measured vs. NNKM model-derived *K*_d_(*λ*) varied from 0.96 to 1.05, while the corresponding determination coefficients varied from 0.72 to 0.92. This was to say that use of NNKM model could account for >72% variations of *K*_d_(*λ*) in optically complex Yellow Sea and China East Sea. These findings implied that the NNKM model did not require further optimization of the weight values of the neural network to accurately estimate the *K*_d_(*λ*) for the testing dataset collected from Yellow Sea and China East Sea, even though the bio-optical properties of this dataset (Table 2) were significantly different from the training dataset collected from the global oceanic and coastal waters (Table 1). Therefore, it may conclude that if properly trained, the NNKM model could yield accurate retrieval of *K*_d_(*λ*) in the global oceanic and coastal waters.

**Table 4:** Performance of SAKM, JNNM, and NNKM models in deriving *K*_d_(*λ*) from testing dataset collected from Yellow Sea and China East Sea during 2003–2012, 174 samples.

Model	Band (nm)	*R* ^2^	*slope*	*bias*	*MRE* (%)
SAKM	443	0.85	1.11	0.009	29.12
	488	0.86	1.12	0.010	29.52
	555	0.82	1.03	0.033	26.92
	667	0.20	0.56	0.244	44.98
JNNM	443	0.52	0.86	0.107	31.32
	488	0.81	0.96	0.033	31.30
	555	0.78	0.77	0.060	27.95
	667	0.62	0.60	0.444	65.26
NNKM	443	0.88	1.01	0.012	28.71
	488	0.92	1.05	0.000	29.17
	555	0.87	0.96	0.015	26.18
	667	0.72	1.05	-0.036	20.19

In order to illuminate the advantages of NNKM model in *K*_d_(*λ*) retrievals, the relationships between SAKM and JNNM models-derived and field-measured *K*_d_(*λ*) also presented here (Table 4 and Fig 4). It was found that the SAKM and JNNM models worked well in deriving *K*_d_(*λ*) from Yellow Sea and China East Sea without further reinitializing the models’ empirical coefficients and/or weights, with the exception of the SAKM and JNNM models-derived *K*_d_(667). Expect 667 nm band, the slopes of linear relationship between field-measured and model-derived *K*_d_(*λ*) varied from 0.77 to 1.12, while the MRE values varied from 26.92% to 31.32%. Use of SAKM and JNNM models could account for >52% variations of *K*_d_(*λ*) at MODIS blue and green bands in Yellow Sea and China East Sea. By comparison, the *K*_d_(*λ*) retrieval accuracy had a significant band changing pattern with values decreasing in the order of 555 nm>488 nm>443 nm>667 nm. These finding implied that the both SAKM and JNNM models could provide an acceptable *K*_d_(*λ*) data at MODIS blue and green bands in Yellow Sea and China East Sea without further optimize the models’ structure and empirical coefficients.

**Fig 5:**
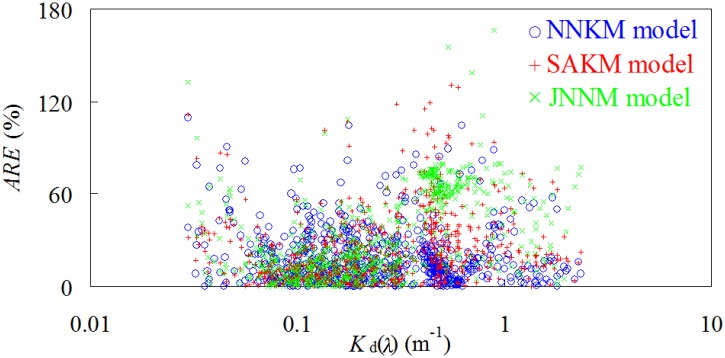
ARE values of *K*_d_(*λ*) predictions plotted versus measured *K*_d_(*λ*) in the Yellow Sea and China East Sea (174 samples).

The *K*_d_(*λ*) was significantly overestimated by SAKM model in the low values, while was clearly underestimated in the high values. By comparison, the NNKM model had a superior performance to both SAKM and JNNM models in deriving *K*_d_(*λ*) from Yellow Sea and China East Sea, especially at 667 nm. Use of NNKM model could decrease respective by 0.41–24.79% and 1.77–45.07% MRE values from SAKM and JNNM models. Judging by MRE values, the models’ retrieval accuracy had a changing pattern with MRE values decreasing in the order of NNKM model>SAKM model>JNNM model. The relationship between ARE and *K*_d_(*λ*) was also presented to demonstrate the ability of the NNKM, SAKM, and JNNM models in estimating *K*_d_(*λ*) in the Yellow Sea and China East Sea (Fig 5). It was found that the ARE value of the NNKM and SAKM models decreased with the increasing *K*_d_(*λ*) value, while the ARE value of JNNM model increased with increasing *K*_d_(*λ*) value, but there is no statistically significant relationship between ARE value and *K*_d_(*λ*). For 0.03 m^-1^<*K*_d_(490)<2.33 m^-1^, the ARE values of the *K*_d_(*λ*) predicted by the NNKM, SAKM, and JNNM models were below 165.99%. When NNKM, SAKM, and JNNM models were applied to data at all bands together the model predicted *K*_d_(*λ*) with a relative random uncertainty of 26.31%, 33.22%, and 41.85%, respectively. The *K*_d_(*λ*) at range of 0.1–1.0 m^-1^ contributed greatly to MRE. These findings implied that all of the NNKM, SAKM, and JNNM models could be used to retrieve *K*_d_(*λ*) from shelf seas, but the NNKM model (*MRE* = 26.31%) had a superior performance in comparison to both SAKM (*MRE* = 33.22%) and JNNM (*MRE* = 41.85%) models. Using the NNKM model for retrieving *K*_d_(*λ*) in the Yellow Sea and East China Sea decreased by 6.92% and 15.55%, respectively, from SAKM and JNNM models.

### Derive *K*_par_ from NNKM model-derived *K*_d_(*λ*)

#### NKKM model initialization

**Fig 6:**
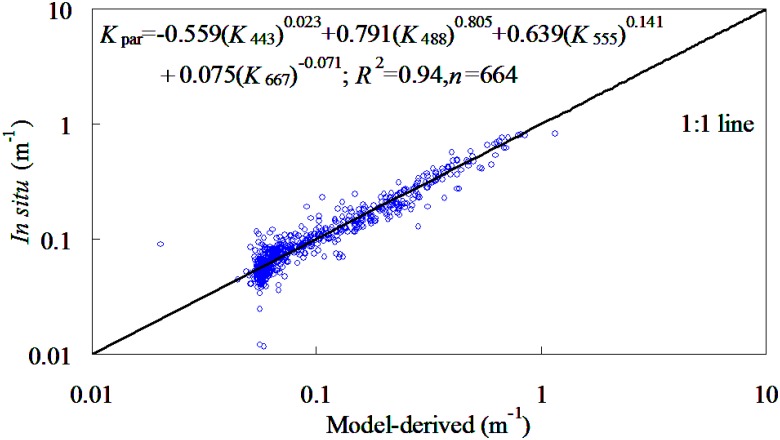
Scattering plots of NKKM model-derived versus field-measured *K*_par_ for NOMAD dataset.

Most optical satellite sensors have calibration problems at 412 nm and near-infrared bands and/or performance of atmospheric correction was in these part of the spectrum [17, 42]. In order to improve the practicability of *K*_par_ retrieval using ocean color satellite, the 412 nm band and near-infrared bands were avoided to use for construction of NKKM model. Based on the synchronous field-measured *K*_par_ and NNKM model-derived *K*_*λ*_, the recursive procedures proposed by Chen and Quan [44] were used to determine the optimal coefficients of NKKM model, which must have the minimum MRE value. Based on 664 samples provided by NOMAD dataset, the NKKM as shown in Fig 6 was proposed as the optimal NKKM model in quantifying *K*_par_ from the global oceanic and coastal waters. It was found that the NKKM model was an effective predictor in retrieving *K*_par_ with MODIS spectral bands, whose determination coefficient was 0.94. This was to say that use of NKKM model could account for 94% variations of *K*_par_ for the NOMAD dataset. Therefore, it may conclude that the NKKM model may be able to provide accurately estimation of *K*_par_ for the global oceanic and coastal waters.

#### NKKM model evaluation

**Fig 7:**
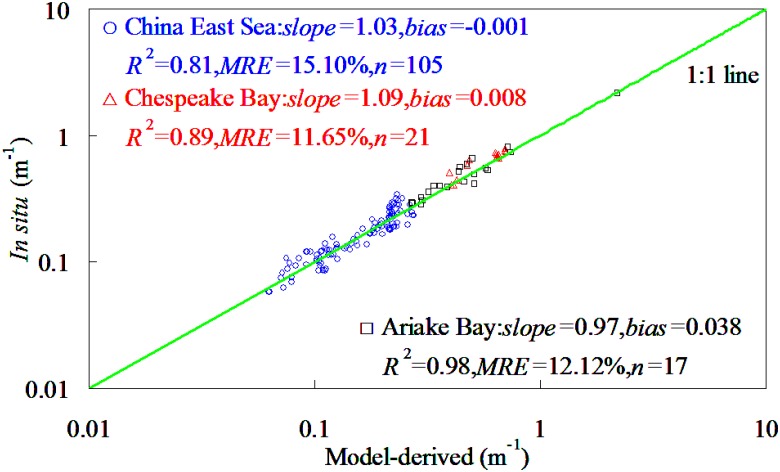
Accuracy evaluation of NKKM model in China East Sea, Chesapeake Bay, and Ariake Bay, respectively.

Here, we present the evaluation of the performance of NKKM model with MODIS spectral bands. The evaluation was based on comparison of the *K*_par_ predicted by NKKM model with *K*_par_ measured analytically for three independent datasets (Table 3). The comparison of the measured and predicted estimates of *K*_par_ by NKKM model was presented in Fig 7. It was found that the NKKM model-derived *K*_par_ was agreed well with the corresponding field measurements. For *K*_par_ ranging from 0.058 to 2.176 m^-1^, the ARE values of *K*_par_ prediction did not exceed 32.06%. The slopes of linear relationships between model-derived and field-measured *K*_par_ varied among the datasets from 0.97 to 1.09, while the corresponding determination coefficients varied from 0.81 to 0.97. This was to say that use of NKKM model could account for >81% variations of *K*_par_ in China East Sea, Chesapeake Bay, and Ariake Bay. The NKKM model predicted *K*_par_ with a relative random uncertainty across the datasets from 11.65% to 15.10%. When it was applied to all four independent dataset in Table 3 together, the model predicted *K*_par_ with a relative random uncertainty of 20.17%. These findings implied that the NKKM model did not require further optimization of mode structure to accurately derive *K*_par_ in bodies with widely varying bio-optical characteristics taken in different regions.

**Fig 8:**
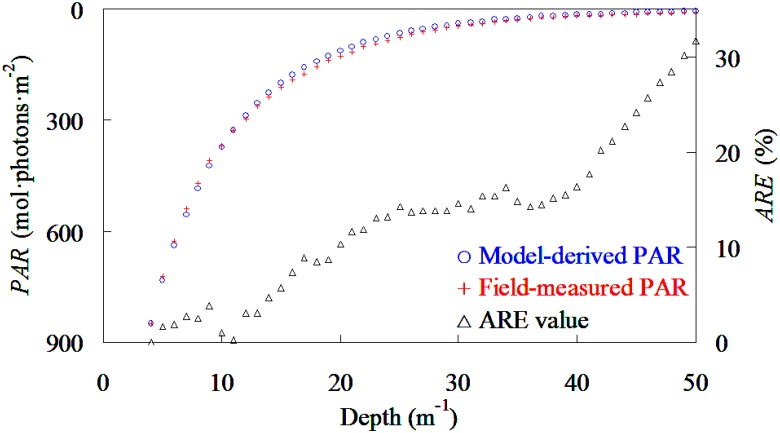
Comparison between the average value of model-derived and field-measured PAR under water based on 75 samples taken from China East Sea.

To further evaluate the stability and accuracy of NKKM model for general oceanographic studies, the profile of PAR values under water taken from China East Seas (75 samples) were calculated using field-measured *PAR*(0) and NKKM model-derived *K*_par_ following Eq (3). Fig 8 showed the comparison between average values of average values of field-measured and model-derived *PAR* under sea waters. As expected, the average values of model-derived agreed well with the field-measured PAR values under water, even though the prediction ARE values increased with the increases of depth which was mainly caused by the wavelength selected absorbing and scattering behaviors of optically activity constituents. Therefore, the NKKM model was an acceptable model in providing model-derived *K*_par_ for oceanographer understanding the vertical variations of PAR under waters.

#### Model comparison

**Fig 9:**
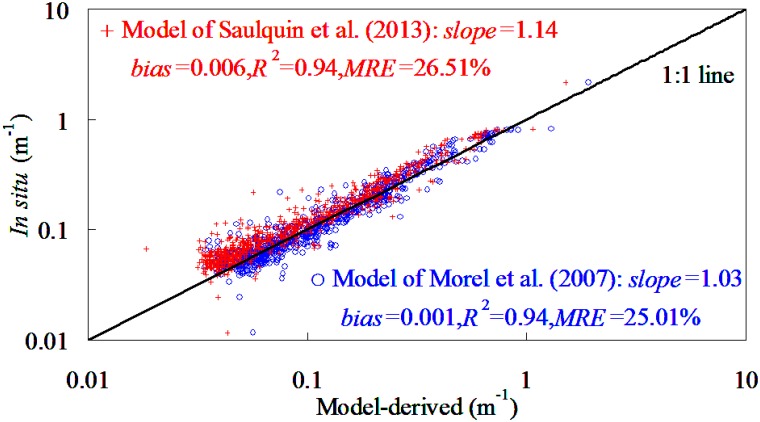
Accuracy estimation of models of Morel et al. [45] and Saulquin et al. [19] using dataset taken in NOMAD, China East Sea, Chesapeake Bay, and Ariake Bay (*n* = 807).

The algorithms of Morel and Saulquin have been described in detail in various references [19, 45]. The measured remote sensing reflectance collected from NOMAD, China East Sea, Chesapeake Bay, and Ariake Bay was fed into NNKM model to generate *K*_490_ data; The derived properties were then fed into the algorithms of Morel et al. [45] and Saulquin et al. [19], and the results were finally compared with the field-measured properties. Following Fig 9, it was found that both Morel and Saulquin models were accurate for global oceanic and coastal waters. When these two models were applied to all four independent dataset together, the models predicted *K*_par_ with a relative random uncertainty of 25.0% and 26.5%, respectively. The *K*_par_ below 0.08 m^-1^ contributed greatly to retrieval uncertainty. The slopes of linear relationship between field-measured and models-derived *K*_par_ were 1.03–1.14, while the corresponding determination coefficients were 0.94. By comparison, the accuracy of NKKM model were comparable with the models of Morel et al. [45] and Saulquin et al. [19]. Use of NKKM model could decrease by 4.83% and 6.34% MRE values from Morel and Saulquin models, respectively. These findings implied that each of NKKM, Morel, and Saulquin models produced acceptable accuracy (*MRE*<27%) in deriving *K*_par_ from both oceanic and coastal waters, but NKKM model worked better than both Morel and Saulquin models.

### Accuracy of satellite-derived *K*_d_(*λ*) and *K*_par_ products

The NNKM, SAKM, and JNNM models-derived *K*_d_(*λ*) data was obtained from the MODIS data after atmospheric correction method proposed by Chen et al. [42]. The accuracy of satellite-predicted *K*_d_(*λ*) was assessed by comparison the satellite-predicted with field-measured results. Nine MODIS imageries synchronizing with in situ measurements in Bohai Sea, Yellow Sea, and China East Sea were collected for use in the experiments of accuracy evaluation. The procedure proposed by Bailey and Werdell [46] was used to generate the satellite-predicted *K*_d_(*λ*) data for match-analysis.

**Fig 10:**
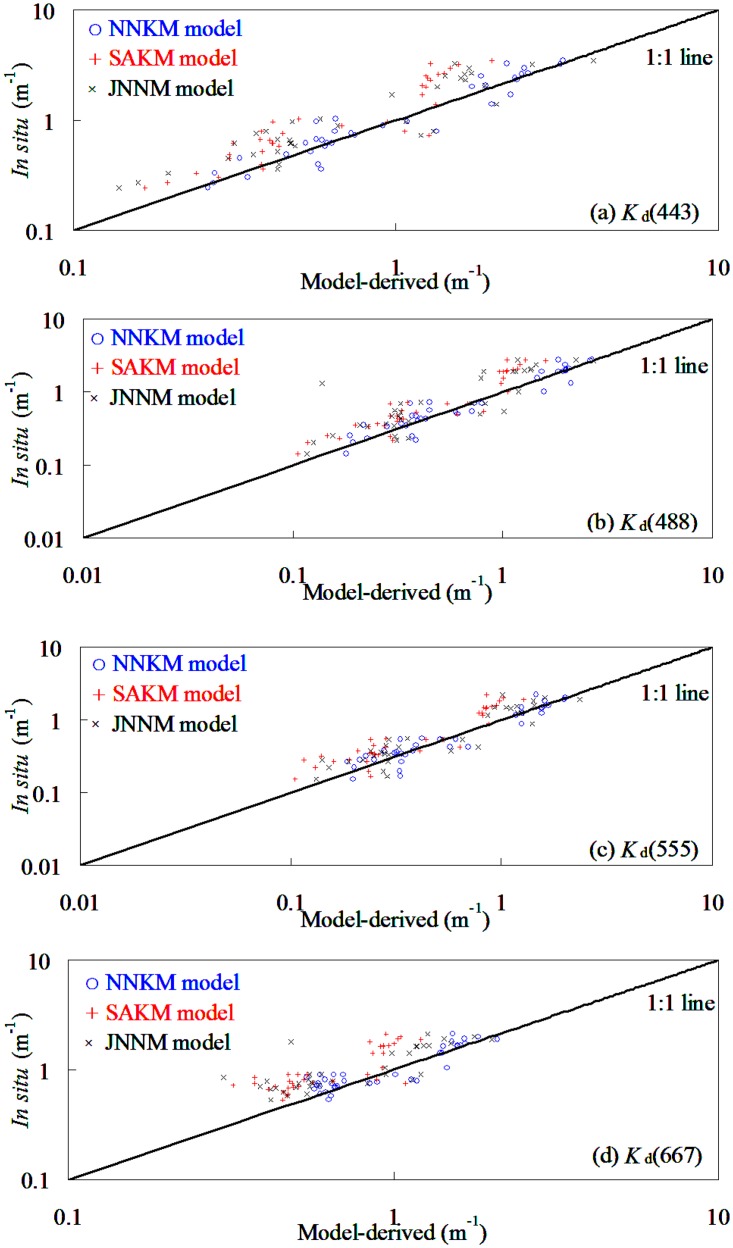
Comparison of the satellite-derived *K*_d_(*λ*) with in situ measurements from Bohai Sea, Yellow Sea, and China East Sea (36 samples).

Fig 10 and Table 5 showed the satellite-derived plotted against the field-measured *K*_d_(*λ*) within a ±3 hour period as the satellite passed over Bohai Sea, Yellow Sea, and China East Sea. It was found that all of the NNKM model produced an acceptable performance in computing *K*_d_(*λ*) from MODIS data. Based on 36 samples extracted from nine different MODIS data, the ARE value of *K*_d_(*λ*) predicted by NNKM model was below 98.83%, with an average of <29.44% of observed *K*_d_(*λ*). The slope of the linear relationships between the NNKM model-derived and field-measured *K*_d_(*λ*) among wavelengths varied from 0.96 to 1.04, while the corresponding determination coefficients were >0.83. This was to say that use of NNKM model could account for >83% variations of *K*_d_(*λ*) in Bohai Sea, Yellow Sea, and China East Sea.

**Table 5:** Comparison of satellite-derived with field-measured *K*_d_(*λ*) in Bohai Sea, Yellow Sea, and China East Sea.

Model	Band (nm)	*R* ^2^	*slope*	*bias*	*MRE* (%)
SAKM	443	0.81	1.80	-0.131	40.14
	488	0.85	1.86	-0.144	38.21
	555	0.86	1.72	-0.068	38.69
	667	0.65	1.54	0.025	38.12
JNNM	443	0.74	1.03	0.283	39.49
	488	0.69	1.17	0.162	40.94
	555	0.78	1.03	0.094	34.32
	667	0.66	0.92	0.322	31.56
NNKM	443	0.91	1.04	0.004	24.59
	488	0.90	1.00	0.029	27.19
	555	0.91	0.99	0.012	29.44
	667	0.83	0.96	0.095	20.94

By comparison (Fig 10 and Table 5), NNKM model (20.94% <*MRE* <29.44) worked better than both SAKM (38.12% <*MRE*< 39.49%) and JNNM (31.56% <*MRE*< 40.94) models in deriving *K*_d_(*λ*) from MODIS data (Table 5). When the NNKM, SAKM, and JNNM models were applied to data at all bands together the model predicted *K*_d_(*λ*) with a relative random uncertainty of 25.57%, 38.80%, and 36.78%, respectively. This was to say that use of NNKM model could decrease by 13.23% and 11.21% MRE values, respectively, from SAKM and JNNM models. These findings implied that all of NNKM, SAKM, and JNNM models could be used to derive *K*_d_(*λ*) from MODIS data in the global oceanic and coastal waters, but NNKM model had a superior performance to both SAKM and JNNM models.

**Fig 11:**
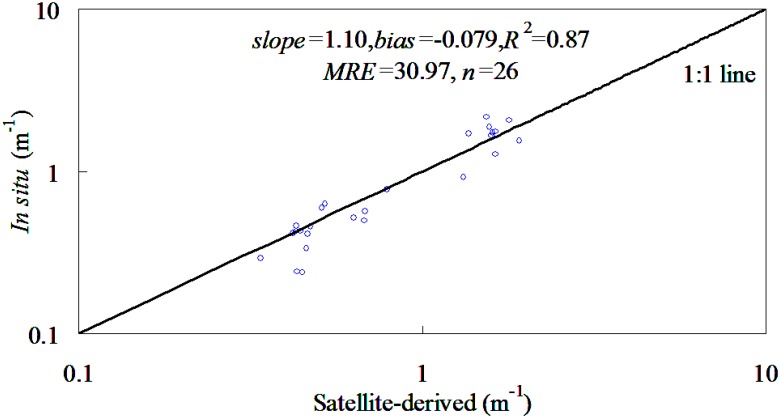
Satellite-derived plotted against NKKM model-derived *K*_par_ in Bohai Sea.

Using the NNKM satellite-derived *K*_d_(*λ*) as the inputs of NKKM model, Fig 11 showed the comparison between the field-measured and model-derived *K*_par_ for independent dataset collected from Bohai Sea during 2005. Based on 26 samples, it was found that NKKM model worked well in deriving *K*_par_ from MODIS data taken from Bohai Sea, whose MRE value was 30.97%. The slope of linear relationship between field-measured and satellite-predicted *K*_par_ was 1.10, while the corresponding determination coefficient was 0.87. This was to say that use of MODIS data with the NKKM model could account for 87% variations of *K*_par_ in the Bohai Sea. These findings implied that, provided that an atmospheric correction scheme for the visible bands was available, the extensive database of MODIS imagery could be used for quantitative monitoring of *K*_par_ in the oceans.

### Spatial and temporal variations of *K*_d_(λ) and Kpar in the global oceanic and coastal waters

**Fig 12:**
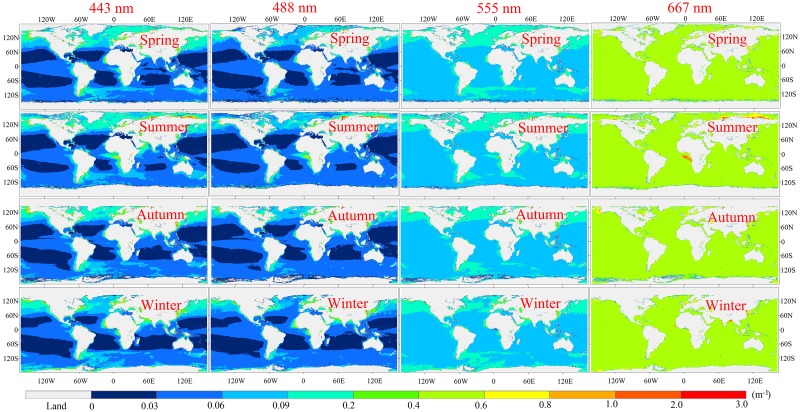
Climatological seasonal mean *K*_d_(*λ*) in the global and coastal oceans (MBPNN-approach, 2002–2013).

The MBPNN model was used to derive a climatological seasonal mean *K*_d_(*λ*) for the global oceanic and coastal waters from the monthly mean MODIS remote sensing reflectance for the time range from July 2002 to September 2013, as shown in Fig 12. It was found that the *K*_d_(*λ*) shows a large variation in the global oceanic waters from 0.0004 to 3.0 m^-1^, with an average value of ~0.055–0.101 m^-1^. The high values, exceeding 1.0 m^-1^, were found in the coastal zones such as the China coastal seas, while the low values of <0.03 m^-1^ were found in open oceans such as the centers of the Atlantic and Pacific Oceans. These high values are caused primarily by land-discharged sediment from inputting rivers, re-suspension of sediment by strong tidal currents, and other factors. Milliman and Meade [47] indicated that rivers with large sediment load contributions of ~7×10^9^ tons of suspended sediment to the ocean yearly, and most of this total is derived from southern Asia. As a result, the coastal zones around southern Asia generally exhibit higher *K*_d_(*λ*) values than other regions, the values around southern Asia ranging from 0.4 to 3.0 m^-1^.

Fig 12 also provides the spectral shape of the diffuse attenuation coefficient as a function of the wavelength in the blue, green, and red regions in the global oceanic and coastal waters. In the coastal regions, the *K*_d_(*λ*) is very high and decreases as a function of the wavelength from blue to the red band with the maximum at 443 nm. The high diffuse attenuation coefficients in the visible regions are most likely due to the high concentrations of colored dissolved organic matters and suspend sediments. In the clear open oceans, unlike the spectral shapes for the coastal zones, the diffuse attenuation coefficient in the blue regions was shown to be very low (<0.03 m^-1^), but becomes higher in the green and red regions, and reached its highest point at 667 nm. By comparison, the diffuse attenuation coefficient in the coastal zones is much higher than the opening oceans.

The cool deepening and warm shallowing of the mixed layer is the principle control of the nutrient supply in the global oceanic and coastal waters [48], which in turn controls the growth rates of primary production in the global oceanic waters [49]. As a result, the higher chlorophyll-a concentration is usually found in the summer [50], due to the fact that the deeper mixed layer reaches down into the higher nutrient waters, bringing a significantly greater amount of nutrients into the mixed layer, which is a result of the higher sea surface temperature in summer [5]. It well known that the optical properties in the global oceanic waters are mainly dominated by the variations of chlorophyll-a concentration [51]. Moreover, most of the global waters belong to the category of oceanic waters. Therefore, the spatial and temporal variations of *K*_d_(*λ*) in the global oceanic and coastal waters depend heavily on the changes of chlorophyll-a concentrations in the open oceans [12]. As a consequence, in the global oceanic and coastal waters summer is the season with the highest *K*_d_(*λ*) values (~0.074–0.101 m^-1^), while winter has the lowest (~0.055–0.088 m^-1^) (Fig 12).

**Fig 13:**
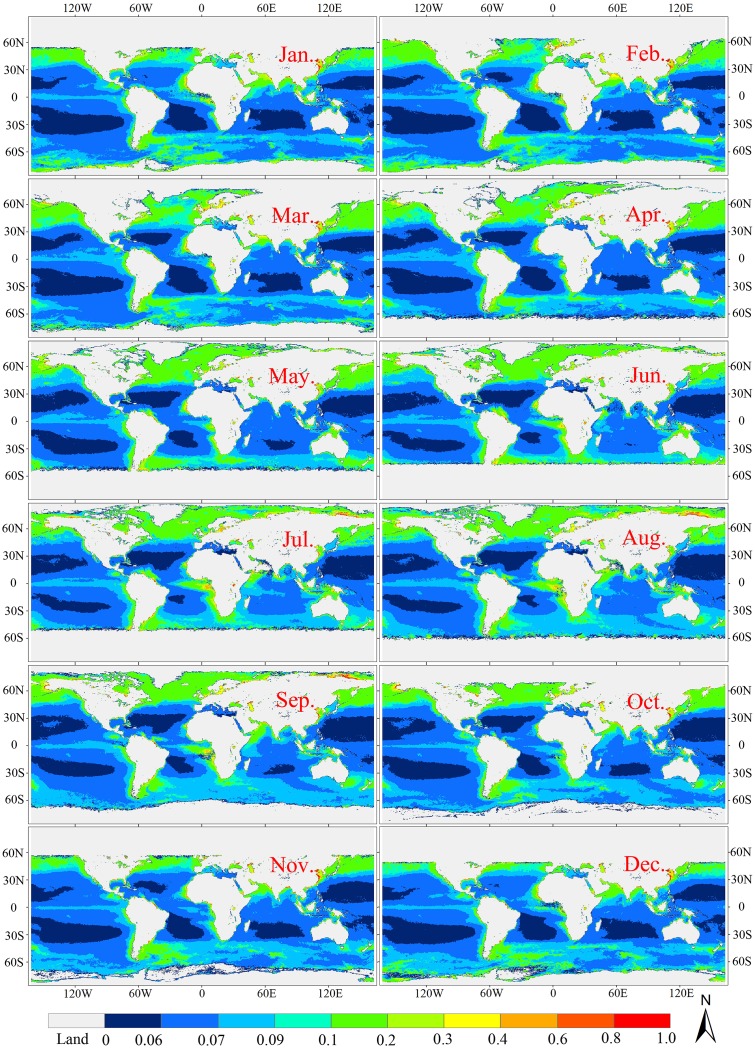
Spatial and temporal variations of *K*_par_ in the global oceanic and coastal waters.

The NKKM model was used to derive climatological monthly mean *K*_par_ for the global oceanic and coastal waters from the monthly mean MODIS remote sensing reflectance for the time ranging from July, 2002 to July, 2015, as shown in Fig 13. As expected, the spatial and temporal variations patterns of *K*_par_ were similar with those *K*_d_(*λ*). For example, the *K*_par_ showed a large variation in the global oceanic and coastal waters, ranging from 0.002 to 14.9 m^-1^; the coastal zone generally exhibited *K*_par_ in the range of 0.3 to 14.9 m^-1^. The width of this zone decreased from south to north with a mean width of about 30 km. The *K*_par_ values from 0.09 to 0.3 m^-1^ were found at the low latitude regions in the Northern Hemisphere, while the centers of Pacific Ocean, Atlantic Ocean, and Indian Ocean were characterized by low *K*_par_ values (<0.06 m^-1^). Moreover, Fig 13 also showed the changing trends of longitude-averaged *K*_par_ values along the latitude in the global oceans, indicating that the *K*_par_ values in the high-latitude areas were much higher than the mid- and low-latitude regions. Moderate *K*_par_ values were found in the equatorial regions of the Atlantic and Pacific Oceans (5°S-5°N), due to the moderate chlorophyll-a concentrations caused by the upwelling of deep, nutrient-rich, cool waters from the divergence of the ocean water masses along the equator [52]. Moderate *K*_par_ values were found in the subtropical convergence zone (around 45°S), where cool, nutrient-rich Sub-Antarctic water masses mix with warm, nutrient-poor subtropical waters [53].

## Discussion

The retrieval accuracy of SAKM model was greatly dependent on the performance of the QAA model. No simplifying assumption can be made that is valid for all special cases existing in the natural world, although the QAA model proved to be robust for deriving inherent optical properties from most global oceanic and coastal waters and some turbid coastal waters [13]. Moreover, another limitation of SAKM model was that the *a*(*λ*) is determined using an empirical model. This empirical approach was able to suppress the effect of *b*_b_(λ) instead of eradicating it completely. As a result, the strong backscattering of suspended particles in turbid coastal waters inevitably exerted a residual effect on the estimation accuracy, which may lead to the violation of QAA model in these waters. As a result, SAKM model may be violated in some special cases, where the bio-optical properties are different from these used for QAA model development. As expect, although the SAKM model-derived was agreed well with the field-measured *K*_d_(*λ*), some limitations still could be found from our studies. For example, the *K*_d_(*λ*) was significantly overestimated by SAKM model in some low values, while was clearly underestimated in some high values. The new *K*_d_(*λ*) model was developed based on the in situ data obtained from the NOMAD, Bohai Sea, Yellow Sea, and East China Sea. As a matter of fact, for this dataset the absorption coefficients for phytoplankton, suspended sediment, and colored dissolved organic matter at the wavelength 490 nm ranges of around from 0.0008 to 1.2488 m^-1^, 0001 to 0.4718 m^-1^, and 0.0001 to 1.4818 m^-1^, respectively. It was found that the variations of optical properties of the dataset collected in this study are quite broad, therefore the NNKM model should be applicable in quite a wide variety of global oceanic and coastal waters.

**Fig 14:**
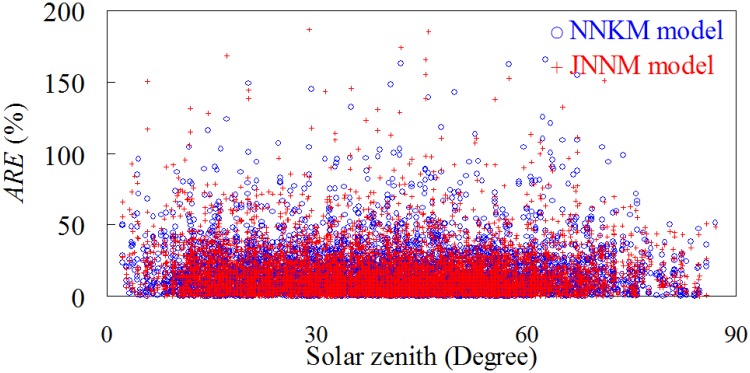
Scattering plots of solar zenith versus ARE values of NNKM and JNNM *K*_d_(*λ*) retrievals.

*K*_d_(*λ*) was an apparent optical water property and thus was dependent on the angular distribution of the underwater radiance distribution [8, 12]. The underwater radiance distribution was altered not only by the absorbing and scattering properties of the water column, but also by effects of variations of the sun zenith angles [54]. As a result, the effects of sun zenith angle on *K*_d_(*λ*) should be taken into account when use the physical models (e.g., models proposed by Aas [29] and Lee et al. [8]) to derive *K*_d_(*λ*) from *a*(*λ*) and *b*_*b*_(*λ*). However, our studies indicated that the *K*_d_(*λ*) could still be accurately (*MRE*<30%) derived from remote sensing reflectance using neural network technology, even though the effects of solar zenith angle was not taken into account during model construction. As a matter of fact, the remote sensing reflectance was an apparent optical property whose value not only depended on inherent optical properties of water column, but also depended on the environmental factors such as solar zenith angle. Using the remote sensing reflectance as the inputs of neural network model, the effects of solar zenith on the accuracy of NNKM and JNNM models-derived *K*_d_(*λ*) could be minimized. Fig 14 showed the relationship between solar zenith angle and ARE value of NNKM and JNNM models-derived *K*_d_(*λ*), indicating that there was no significant relationship between them (*R* ^2^<0.01, *p*<0.05). These findings implied that the effects of solar zenith angle on NNKM and JNNM models’ *K*_d_(*λ*) retrieval accuracy could be neglected, even though the solar zenith and *K*_d_(*λ*) values varied widely in this work.

**Fig 15:**
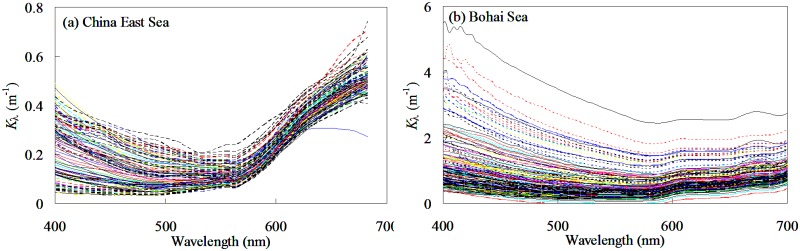
Spectral of diffuse attenuation coefficient from (a) China East Sea and (b) Bohai Sea.

Fig 15 showed the spectral diffuse attenuation coefficients collected from China East Sea and Bohai Sea. It was found that in some samples, the *K*_*λ*_ decreased as a function of the wavelength from blue to green, with the maximum at the blue band and then gradually increased as increase of the wavelength from green to red bands; In other samples, the *K*_*λ*_ values were very low in the blue and green bands, whereas the diffuse attenuation coefficient increased to the red bands due to water absorption. Traditionally, the *K*_par_ was expressed as a function of *K*_490_ [3, 19, 20, 45]. The underlying assumption of traditional *K*_490_-*K*_par_ model was that the diffuse attenuation coefficient at visible bands should be mathematically depended on that at 490 nm, if the left item in Eq (5) could be approximated to the right items. However, no simplified assumption could be made that was valid for all special cases existing in the natural world. As a result, the traditional model would be violated in some optically complex waters where the optical properties were different from these used for model development.

**Fig 16:**
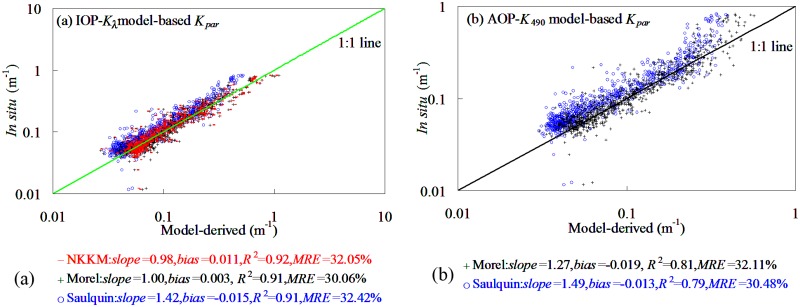
Scatter plots of field-measured versus IOP and AOP models-based models-derived *K*_par_.

Importantly, as the *K*_*λ*_ were the key inputting variables for NKKM model, the performance of *K*_par_ retrieval models strongly depended on the accuracy of retrieval *K*_*λ*_ model. Currently, there were several existing models for computing *K*_*λ*_ or *K*_490_ such as *K*_*λ*_ retrieval model (inherent optical properties (IOP)-*K*_*λ*_ model) proposed by Lee et al. [8] and *K*_490_ retrieval model (apparent optical properties (AOP)-*K*_490_ model) proposed by Mueller [30]. Fig 16 showed the performance of IOP-*K*_*λ*_ and AOP-*K*_490_ models in deriving *K*_par_ from the datasets collected in this study. It was found that combined with IOP-*K*_*λ*_ and AOP-*K*_490_ models, the NKKM, Morel, and Saulquin models produced good performance in deriving *K*_par_ from our datasets, whose MRE values did not exceed 33%. The *K*_par_ below 0.08 m^-1^ and above 0.5 m^-1^ contributed greatly to retrieval uncertainty. By comparison, the neural network model-based KNNM, Morel, and Saulquin models (20.17%<*MRE*<26.51%) produced a superior performance to IOP-*K*_*λ*_ and AOP-*K*_490_ models-based KNNM, Morel, and Saulquin models (30.06%<*MRE*<32.42%). The reasons to these results may be that the performance of neural network model produced a superior performance to IOP-*K*_*λ*_ and AOP-*K*_490_ models in deriving *K*_*λ*_ from the global oceanic and coastal waters. Therefore, in order to accurately retrieve *K*_par_ from the global oceanic and coastal waters, the *K*_*λ*_ fed to the procedures of the NKKM model should be accurately enough.

Finally, the calibration and validation datasets used in this study were taken from the NOMAD, China East Sea, Ariake Bay, and Chesapeake Bay. Although the variations of optically active constituents are quite wide and therefore the NKKM model should be applicable in quite a wide variety of global waters, it may be still insufficient to completely validate the accuracy of the NKKM model in other waters with different bio-optical properties found throughout the world. Thus, it is concluded that the NKKM model should be used for estimating *K*_d_(*λ*) and *K*_par_ in the global oceanic and coastal waters, but some further works about the model calibration and validation with different dataset was still desired in the future.

## Summaries

In this study we have proposed an approach to monitor the instantaneous diffuse attenuation coefficients and application to monitor the spatial and seasonal variations of *K*_par_ in the global oceanic and coastal waters from space. The major difference between this study and previous reports lies in that the spectral *K*_d_(*λ*) in the visible regions is estimated directly from the high temporal and spatial coverage of water-leaving apparent optical properties offered by the MODIS satellite. Moreover, the performance of our model was compared with that of SAKM and JNNM models in deriving *K*_d_(*λ*) from global oceanic and coastal waters. The SAKM and JNNM models produce an acceptable accuracy for retrieving *K*_d_(*λ*) at blue and green wavelengths, but had a poor performance in deriving *K*_d_(*λ*) from in the global oceanic and coastal waters. In comparison to SAKM and JNNM models, our model produces a superior performance in estimating *K*_d_(*λ*) from global oceanic and coastal waters. These studies have proven that the *K*_d_(*λ*) model based on artificial neural network technology is a practical method for processing satellite remote sensing data, although models using analytical semi-analytical approaches may map *K*_d_(*λ*) distribution more efficiently.

Solar radiation available for photosynthesis regulated primary productivity, or the rate of carbon fixed by marine ecosystems. The solar radiation penetration and availability in aquatic systems could be expressed by *K*_par_, which is defined in terms of the exponential decrease of the ambient irradiance with depth. This work made a contribution to the ocean optical community by providing improved capacity to retrieve *K*_par_ from global oceanic and coastal waters. The authors have evaluated the performances of two existing *K*_par_ retrieval model models for the datasets collected from NOMAD, China East Sea, Ariake Bay, and Chesapeake Bay, and further improved these for global oceanic and coastal waters using a NKKM model. The study results indicate that all three models could provide an acceptable accuracy in quantifying *K*_par_ from global oceanic and coastal waters, but NKKM model produced a superior performance from two existing models.

The *K*_d_(*λ*) and *K*_par_ were quantified from MODIS images after atmospheric correction using a NIR-based and SWIR-based combined model. After comparison between the satellite-derived and field-measured *K*_d_(*λ*) and *K*_par_, it was seen that our model produces <31% MRE values in deriving *K*_d_(*λ*) and *K*_par_ from global oceanic and coastal waters. Moreover, the ARE values of NNKM model-derived *K*_d_(*λ*) were independent on the solar zenith angle, because using the apparent optical properties such as remote sensing reflectance as the inputs of neural network model, the effects of solar zenith on the retrieval results had been minimized. These studies provide important insight for improving ocean color models and bio-optical models, as well as for more accurate retrieval of *K*_d_(*λ*) and *K*_par_ in the global oceanic and coastal waters. Finally, our model is also proposed to retrieve the global climatological seasonal mean *K*_d_(*λ*) for the time range from July 2002 to September 2013. Due to the effects of river-discharged suspended sediments, the *K*_d_(*λ*) and *K*_par_ around the coastal zones is always higher than that in the oceanic waters. Due to the seasonal variations of chlorophyll-a concentration, the global mean *K*_d_(*λ*) and *K*_par_ in the summer is usually higher than that in the winter. These results were advantage for improving our knowledge about the light field under waters at the global and basin scale.

## Supporting Information

S1 File The compute code of Kd spectral retrieval model developed in this study. (RAR)
